# Medication Adherence to Intranasal Corticosteroids in Allergic Rhinitis Patients with Comorbid Medical Conditions

**DOI:** 10.3390/pharmaceutics14112459

**Published:** 2022-11-15

**Authors:** Prempreet Kaur Manjit Singh, Elang Kumaran Krishnan, Norhafiza Mat Lazim, Najib Majdi Yaacob, Baharudin Abdullah

**Affiliations:** 1 Department of Otorhinolaryngology-Head and Neck Surgery, School of Medicine Sciences, Universiti Sains Malaysia, Kubang Kerian 16150, Malaysia; 2 Department of Otorhinolaryngology-Head and Neck Surgery, Hospital Kuala Lumpur, Jalan Pahang 50586, Malaysia; 3 Unit of Biostatistics and Research Methodology, School of Medical Sciences, Universiti Sains Malaysia, Kubang Kerian 16150, Malaysia

**Keywords:** allergic rhinitis, intranasal corticosteroids, medication adherence, comorbid medical diseases, adverse effects, Brief Medication Questionnaire, immunoglobulin E

## Abstract

Background: To determine medication adherence to intranasal corticosteroid spray (INCS) among allergic rhinitis (AR) patients with comorbid medical conditions. Methods: A cross-sectional study was conducted. Adults above 18 years old with persistent symptoms of AR and comorbid physician-diagnosed asthma, eczema, diabetes mellitus (DM) and hypertension (HPT) were included. The severity of symptoms was assessed by the total nasal symptom score (TNSS), medication adherence was based on the patients’ diaries and barriers to adherence were analyzed by the Brief Medication Questionnaire. Results: 185 participants were enrolled. The medication adherence was 58.9%. Medication adherence was significantly superior in participants with elevated total serum immunoglobulin E (IgE) (χ2 = 8.371, *p* < 0.05), house dust mite (HDM) allergy to *Dermatophagoides pteronyssinus* (DP) type (χ2 = 5.149, *p* < 0.05) and severe TNSS at the first visit (χ2 = 37.016, *p* < 0.05). Adherence was twice more likely in DP allergy, 2.7 times more likely in elevated total IgE and 15 times more likely in severe TNSS at the first visit. Among the barriers to adherence was lack of symptoms, taking medication only when necessary, fear of adverse effects, running out of medication, experiencing bothersome effects, ineffective response, forgetfulness and taking too many medications. Only lack of symptoms, taking medication when symptomatic, fear of adverse effects and running out of medication were significant. No significant association was found between asthma/eczema (χ2 = 0.418, *p* > 0.05), HPT/DM (χ2 = 0.759, *p* > 0.05) and multi-medicine use (χ2 = 1.027, *p* > 0.05) with medication adherence. Conclusions: Patients having AR with severe nasal symptoms at first presentation, who are sensitized to DP HDM and who have elevated total serum IgE levels have a higher adherence to INCS use. The use of multiple medicines had no impact on the adherence to INCS. As a lack of symptoms was a barrier towards adherence, the benefits of using INCS according to the prescribed dose and frequency must be emphasized to patients with mild and moderate AR at each medical visit. A good rapport between patients and their health care providers is needed to build trust and overcome the barriers, particularly to allay the fears of adverse effects of INCS. The other barriers, such as running out of supply, can be overcome by posting medications directly to patients by the healthcare providers.

## 1. Introduction

Intranasal corticosteroids (INCS) are the recommended first line pharmacotherapy in moderate to severe allergic rhinitis (AR) due to its efficacy and effectiveness in controlling inflammation and overcoming the symptoms of AR, particularly nasal congestion [1,2]. The negative impact on the quality of life of patients with uncontrolled AR is well acknowledged. Nasal obstruction contributes to sleeping disturbances leading to tiredness and cognitive impairments such as poor focus and attention span. Moreover, persistent sneezing and rhinorrhea give rise to the need of keeping tissue paper always at hand, which can affect the self-esteem and social standing of the sufferers. Medication adherence is defined as the process by which patients take their medications as prescribed, composed of initiation, implementation and discontinuation phases [3,4]. Medication non-adherence can occur at any stage along this integrated process. Factors affecting adherence can be divided into five different dimensions: social and economic factors, therapy-related factors, disease-related factors, patient-related factors and health care system-related factors [5]. Patient-related factors include unintentional factors for example forgetting to take medication or inadequate understanding of dose or schedules, and intentional factors involve an active decision to stop or modify a treatment regimen based on ability to pay, beliefs and attitudes about their disease, medication side effects, and expectations for improvement [6]. Examples of the non-modifiable factors are gender, age and level of education while the medication beliefs and medication regimens are modifiable factors.

Adherence to medications plays a critical role in accomplishing good outcomes in chronic airway conditions such as AR, bronchial asthma and chronic obstructive pulmonary disease. In spite of this fact, it has been reported that adherence to treatment in allergic airway disease was insufficient where less than half of medications prescribed was taken [7]. Similarly, non-adherence to INCS is suboptimal which results in an inadequate and unsatisfactory control of AR. Though the contributing factors to non-adherence are multifactorial, one of them is doubts over medication-related factors, primarily their effectiveness and concerns of adverse effects [8]. Additionally, disruption of daily activities by the medication regimens was inferred. These problems could escalate in patients having allergic disorders and comorbid medical conditions as adherence tends to be affected when patients are prescribed multiple medicines at the same time [9]. In the treatment of AR, patients must use the INCS topically by spraying their noses as compared to the oral intake of medications for their other medical conditions. The different administration system for dissimilar form of medications could potentially create a problem in adherence.

The purpose of this study was to assess medication adherence to INCS and the related barriers in the treatment of AR patients with comorbid medical conditions.

## 2. Materials and Methods

### 2.1. Study Design and Setting

The study was conducted in accordance with the Declaration of Helsinki, and ethical approval was obtained from the Medical Research and Ethic Committee of National Medical Research Registry (NMRR) Malaysia [NMRR-19-3955-52224]. A cross-sectional study was carried out at the otorhinolaryngology clinic of a tertiary hospital in Kuala Lumpur from January 2020 until June 2021. Informed consent was obtained from all patients prior to recruitment. A convenient sampling method was used to recruit all patients with AR that fulfilled the selection criteria. Consenting patients were given the proforma form to document their allergy history and other pertinent information. Supplementary Table S1 shows the complete particulars in the proforma. After completion of the required assessments, they were scheduled for follow-up appointments at the 4th and 12th week from the first visit.

### 2.2. Participants

Adults above 18 years old having persistent symptoms of AR according to Allergic Rhinitis and its Impact on Asthma (ARIA) guidelines [1] with or without comorbid physician-diagnosed chronic medical conditions, specifically asthma, eczema, diabetes mellitus (DM) and hypertension (HPT), were included. All patients were prescribed INCS sprays once daily for the whole study duration. The assessment of medical co-morbid conditions was based on a documented hospital record of an established diagnosis by physician and existing prescriptions or medications. Patients with comorbid medical conditions must be on medications prescribed by physicians for at least one year. Patients with underlying sinusitis, nasal polyposis, nasal septal deviation, sinonasal malignancy and recent nasal surgery (less than 5 years) were excluded. The sociodemographic information, medical and allergy history were recorded. Included in the sociodemographic characteristics were marital status, as well as the number of children and pets kept at home. The level of education was based on the International Standard Classification of Education and divided into primary, secondary and tertiary education [10]. The occupation was classified according to professional or non-professional together with the duration of working hours. Professional occupation was defined as a job that requires a Bachelor, Master or Doctor of Philosophy degree [11]. Examination was performed by nasoendoscopic examination and allergy status was confirmed by skin prick or serum specific immunoglobulin E (IgE) test. A positive skin prick test (SPT) result was defined as a wheal of more than 3 mm to allergens with a nonreactive negative control after 15 min. A value of 0.35 kU/L or more of the serum specific IgE was considered positive for allergy. Patients were given a medicine diary to document medication intake and record adverse effects. Among the adverse effects appraised were those associated with the use of INCS such as pain, epistaxis and aftertaste [12]. All patients were taught the correct application of the INCS and were requested to demonstrate the technique at least once to ensure proper nasal spray administration. 

### 2.3. Asessment of Outcomes

#### 2.3.1. Assessment of Symptoms

The severity of symptoms was determined by the total nasal symptom score (TNSS). The TNSS is a reliable and valid test for evaluation of nasal symptoms in patients with AR [13]. The TNSS comprises the sum of score for nasal congestion, sneezing, nasal itching and rhinorrhea with each using a 4-point scale of 0 to 3. A score of 0 indicates no symptoms, a score of 1 indicates mild symptoms, 2 for bothersome symptoms but tolerable and 3 being severe symptoms that are hard to tolerate and interferes with daily activities. A score of less than 6 was graded as mild, score of 6 to 9 moderate and score of 9 to 12 severe TNSS. The first assessment was carried out at the first visit and subsequently at four and twelve weeks following enrollment. The total serum IgE level at the first visit was measured. All patients were given INCS and were counselled on the dosing and proper technique of application. During follow up visits, the adherence was assessed in addition to the severity of symptoms, findings on examination and the technique of using the nasal spray.

#### 2.3.2. Assessment of Adherence

The implementation phase of adherence was assessed. Adherence to medication was based on the diary given to the patients by calculating the number of days patients used the nasal spray (total number of days’ use of medication/prescription period × 100). Adherence was defined as taking 80% or more of the prescribed medication [14]. Supplementary Figure S1 shows the patients’ diary.

#### 2.3.3. Assessment of Barriers to Adherence

The challenges faced by patients in adhering to treatment were evaluated by the Brief Medication Questionnaire (BMQ) at the 12th week by direct interview (first author). The BMQ is sensitive, brief and has the ability to discover different types of non-adherence, identify specific domains of barriers and evaluate multi-medicines intake [15,16]. The BMQ is a validated and reliable tool for measuring adherence to medications in patients with chronic health conditions [17]. It is divided into three main sections, specifically the ‘Regimen’, ‘Belief’ and ‘Recall’. The 5-item ‘Regimen’ asks about the medication intake including the name and strength of the prescribed medication, frequency, doses, concurrent medication intake and missed doses, the 2-item ‘Belief’ addresses medicine efficacy and disturbing effects from medication intake, and the 2-item ‘Recall’ addresses remembering difficulties, particularly difficulty in taking multiple medications and trouble in remembering to take the medications. We defined multiple medicines as the use of more than 3 medicines based on a study done by Arnet et al. [18]. The score for the ‘Regimen’ section ranges from 0 to 7, and the ‘Belief’ and ‘Recall’ sections both range from 0 to 2. The maximum total BMQ score is 11 and any score greater than zero for any one of the sections indicates potential non-adherence to prescribed treatment.

### 2.4. Statistical Analysis

Data were analyzed using IBM Statistical Package for Social Science (SPSS) version 25. Descriptive statistics were used to describe characteristics of the participants and expressed as mean ± SD for numerical and frequency (%) for categorical variables. A Chi-squared test (χ2-test) was used to measure associations between categorical variables and medication adherence. The significant associated factors were tested by simple logistic regression to identify variables associated with the medication adherence. *p* < 0.05 was considered to be statistically significant.

## 3. Results

### 3.1. Characteristics of Study Participants

Of the 200 eligible participants approached, 10 declined participations. 190 participants were recruited with 185 participants completed the study and 5 patients dropped out. The age of participants ranged from 18 to 74 years old, with a mean of 37.9 ± 14.8 years. The sociodemographic characteristics are shown in Table 1. The majority of participants had tertiary education (55.7%) and non-professional jobs (61.1%) with less than 42 h of work per week (62.2%) (Table 2). Most of the participants reported a history of allergy to food and/or aeroallergens (69.7%) but only 31.4% had a positive family history of allergy. The sensitization to allergens and levels of IgE are shown in Supplementary Table S2. The distribution of comorbid medical conditions and the use of multiple medicines (more than three medicines) is shown in Table 3. The patients were using only INCS and not on any other systemic treatment for AR. The duration of AR and use of INCS are shown in Table 1. Anti-hypertensive agents were given for HPT, oral hypoglycemic agents with or without insulin for DM, inhaled salbutamols with or without inhaled corticosteroids for bronchial asthma and topical steroids cream for eczema. Supplementary Table S3 shows the severity of TNSS during the first, second and third visits.

### 3.2. Use of INCS

A total of 54.6% participants received budesonide, 25.9% mometasone furoate and 19.5% fluticasone furoate nasal sprays (Supplementary Table S2). There was no significant difference seen in the medication adherence (χ2 = 4.122, *p* > 0.05) between the nasal sprays among participants.

### 3.3. Factors Associated with Adherence

The medication adherence to INCS was 59.5%. Table 1 shows the association between sociodemographic factors and medication adherence to INCS. There were no significant differences between medication adherence and age (χ2 = 0.67, *p* > 0.05), gender (χ2 = 2.72, *p* > 0.05), marital status (χ2 = 4.64, *p* > 0.05), number of children (χ2 = 0.488, *p* > 0.05) and keeping pets at home (χ2 = 0.084, *p* > 0.05). No significant association was found between medication adherence and known allergies (χ2 = 0.052, *p* > 0.05), family history of allergy (χ2 = 1.27, *p* > 0.05), asthma and eczema (χ2 = 0.418, *p* > 0.05), hypertension and diabetes mellitus (χ2 = 0.759, *p* > 0.05) and multi- medicine use (χ2 = 1.027, *p* > 0.05).

Though higher prevalence in adherence was seen in participants who were professionals (65.3%) and had tertiary education (64.2%), there were no significant association between medication adherence with level of education (χ2 = 5.76, *p* > 0.05), occupation (χ2 = 2.46, *p* > 0.05) and working hours (χ2 = 0.037, *p* > 0.05). 

Medication adherence was significantly greater (χ2 = 8.371, *p* < 0.05) in participants having elevated total serum IgE levels, rather than normal levels. Sensitization to house dust mite (HDM), particularly *Dermatophagoides pteronyssinus* (DP), played a significant role in medication adherence (χ2 = 5.149, *p* < 0.05). Despite the higher medication adherence in participants with seafood, cockroach and cat dander allergy, the association was not significant. The severity of TNSS was significantly associated with the medication adherence during the first (χ2 = 37.016, *p* < 0.05) and second visit (χ2 = 10.304, *p* < 0.05) but not at the third visit (Supplementary Table S3).

### 3.4. Post-Hoc Analysis

A simple logistic regression test showed a significant difference in the severity of TNSS at the first visit with the medication adherence (*p* = 0.001), but the severity of TNSS in the second visit was not significant (*p* = 0.217) (Table 4). Participants with DP allergy were twice more likely to be adherent to INCS than participants without the DP allergy (OR = 2.13, 95% CI:1.10–4.11, *p* = 0.001), while participants who had elevated total IgE were 2.7 times more likely to be adherent to INCS when compared to those with normal levels (OR = 2.69, 95% CI: 1.36–5.34, *p* = 0.004). Those with severe TNSS during the first visit to the clinic were 15 times more likely to be adherent to INCS compared to mild TNSS (OR = 15.13, 95% CI: 5.69–40.22, *p* = 0.001).

### 3.5. Barriers to Adherence

Table 5 shows the association between the ‘Regimen’, ‘Belief’ and ‘Recall’ sections of BMQ with medication adherence. Only ‘Regimen’ has a significant association with medication adherence. The barriers for non-adherence are shown in Table 6. Among the reasons given in the ‘Regimen’ section were a lack of symptoms, taking medication only when needed, especially when the symptoms worsened, and missed doses on account of fear of adverse effects and running out of medication supply. In the ‘Belief’ section, barriers to adherence were experiencing bothersome effects and ineffective responses to INCS use. Bothersome effects of the medications such as sneezing, worsening nasal obstruction, aftertaste and feeling dizzy after using the nasal spray were disclosed as the pretext for non-adherence regardless of the type of nasal sprays. Meanwhile, for the ‘Recall’ section, participants cited a busy schedule and forgetfulness as the reasons for not using INCS. One participant mentioned taking too many medications as the reason for not adhering to INCS.

## 4. Discussion

### 4.1. Main Findings

Allergic rhinitis affects a large proportion of the population throughout the world, including Asia, with its prevalence ranging from 27% in South Korea to 32% in the United Arab Emirates [19]. The main treatment of AR involves treatment by INCS but the adherence to its use remains problematic with diverse rate across the countries worldwide. Compared to a study done in Italy which had an adherence to INCS of 85.7%, the 59.5% adherence rate found in our study is considerably lower, but it is comparable to a study from Singapore (63.1%) [12,20]. 

The prevalence of a concomitant bronchial asthma in allergic rhinitis patients ranged from 28% to 67.3% in other studies [21,22,23]. However, we found our patients had lower prevalence (18.9%). The environmental differences and allergic triggers could account for this discrepancy. In the present study, concomitant DM, HPT, bronchial asthma and eczema did not affect medication adherence. 

Elevated total serum IgE was a common finding in allergic conditions, which can help clinicians to verify the diagnosis of bronchial asthma or allergic rhinitis. A higher total serum IgE level is linked with a greater burden of disease exemplified in a study by Nukhbat et al., who found that serum IgE levels were higher in moderate to severe AR compared to the milder form [24]. The majority of our patients had an elevated total serum IgE and it is associated with a significantly higher medication adherence.

Notably, the most common allergen seen among participants in this study was the HDM DP type. This prevalence is similar to the finding of a study from Thailand, which showed that 75% of patients were positive to HDM DP [25]. A significant association was demonstrated between the presence of DP allergy with medication adherence to INCS. Participants with a positive SPT to DP were inclined towards better medication adherence than participants with negative DP.

Non-adherence to prescribed medicine regimens poses a significant risk for decreased therapeutic effects or failure, irrespective of the disease or patient characteristics [26]. In patients with multiple medical conditions requiring the use of multiple medicines, non-adherence could potentially occur [27,28]. Remarkably, the multiple administration of different forms of medications in our study consisting of solid oral medicines, inhalers and intranasal sprays did not influence adherence. Clearly, this showed that non-adherence arises due to a combination of factors and not just due to a single factor such as the use of multiple medicines. In spite of the use of multiple medicines, a study [29] demonstrated that patients had high medication adherence when they believe in the benefit of adhering to the prescription, as well as had fear of the consequences when they did not. Trust towards health-care professionals also was stated to be the reason for the high adherence [29].

Understanding the barriers to adherence is one aspect of improving the medication adherence. In this study, we have identified several barriers to adherence. Non-adherence in chronic conditions occurs commonly when there is a lack of symptoms. It is apparent that severe symptoms at presentation in AR patients ensure strong medication adherence as patients who want to improve their quality of life. When the symptoms have subsided, the majority of patients will discontinue their medication as they are under the impression that the problem has resolved. Experience of adverse effects from using previous medications or seeing friends or family member taking medications that have them caused patients to have a fear of potential effects with continuous use of their medications. This can lead to patients missing the required dose or reducing the frequency of the dosing by using it on alternate days or at a longer interval. Thus, it is imperative that patients are well-acquainted with the necessity of the medications, the expected outcomes from the treatment and the nature of the adverse effects of each specific medication to circumvent non-adherence. A study has highlighted that medication adherence is facilitated through countering doubts about personal need and addressing treatment concerns of patients by the prescribers [30].

The prescription of different forms of medication requiring disparate route of intake could increase the chances of non-adherence. This becomes more pronounced especially in the presence of other factors such as a busy schedule and concerns of medicine interactions. 

An experience of bothersome effects from the INCS caused patients to fear using it continuously. Along with these effects, they may feel that the treatment is ineffective. This will culminate in patients not taking the prescribed the INCS or taking it only when necessary. The other factor that could make patients not taking the prescribed medications in the first place or taking them only when necessary is cost. The cost of the medicine given to the patient often forms the major barrier to adherence. As the present study was conducted in a public hospital, which is backed financially by the government where medications are subsidized and inexpensive, the issue of cost did not arise.

In the present study, only the ‘Regimen’ section significantly affected the medication adherence. Of note, a study evaluating medication adherence in chronic medical disease had reported that the ‘Regimen’ impacts non-adherence more than the ‘Belief’ and ‘Recall’ sections [31].

Medication adherence could be shaped by several factors, including individual factors (i.e., socioeconomic status, age, sex, and race) and health system factors (i.e., health literacy, the convenience of pharmacies, and medication regimen complexity) [32]. Individual factors consist of modifiable and non-modifiable barriers. Modifiable barriers such as patient beliefs and concerns of adverse effects may lead to non-adherence and should be adequately addressed. The collaboration of pharmacists and other healthcare professionals has been effective in simplification of complex regimens to improve adherence and clinical outcomes [33]. Nonetheless, medication adherence needs to be seen as a multidimensional construct. As adherence is a process enacted over time, the riposte should be more wide-ranging [4]. Patients, health care providers, and health care systems all have a role to improve medication adherence. A single method cannot improve medication adherence; instead, a combination of various adherence techniques should be implemented to improve patients’ adherence to their prescribed treatment. Thus, efforts to improve rates of adherence often must incorporate multiple strategies across the continuum of care [34].

### 4.2. Strengths and Weaknesses

Data on adherence to INCS from South East Asian countries are scarce. Our findings enrich the knowledge and understanding of the patterns of adherence in AR patients from this particular region. Another highlight of our study is the study population that consisted of AR patients with co-morbid medical conditions. When treating patients with AR, we often encounter patients having several other medical conditions at the same time. The use of multiple medications to treat these health conditions can lead to problems with adherence. With this in mind, the present study provides a real-world evidence of medication adherence.

The use of subjective measures can be construed as the main weakness of this study. However, it must be reiterated that there are no ideal methods to accurately record medication adherence. Measures such as patient-kept diaries, patient interviews and questionnaires such as BMQ are adequately proficient to evaluate the adherence level. These methods offer a cost-effective option in a resource limited setting and convenient to be used even in large populations. They are valuable to determine the concerns and worries of patients while undergoing treatment in an effort to overcome barriers to the medication adherence.

We acknowledge the small sample size in this study might not be able to detect the difference between the two groups and the results could be accidental. Considering the innumerable number of people suffering from AR, the number of participants of 185 in this study might not be representative of the actual adherence-related issues of this condition. The change of threshold of 80% as medication adherence could also potentially alter the results. The threshold of 80% as medication adherence is established with cardiovascular disease and highly controversial. Therefore, the results in this study must be interpreted with caution. Future studies with a higher number of patients and different threshold for medication adherence are necessary.

## 5. Conclusions

The medication adherence to INCS among AR participants in this study was 58.9%. Patients having AR with severe nasal symptoms at first presentation, who are sensitized to DP HDM and who have elevated total serum IgE levels have a higher adherence to INCS use. The use of multiple medicines had no impact on the adherence to INCS. As a lack of symptoms was a barrier towards adherence, the benefits of using INCS according to the prescribed dose and frequency must be emphasized to patients with mild and moderate AR at each medical visit. A good rapport between patients and their health care providers is needed to build trust and overcome the barriers, particularly to allay the fears of adverse effects of INCS. The other barriers, such as running out of the supply of medicine, can be overcome by posting medications directly to patients by the health care providers.

## Figures and Tables

**Table 1:** The characteristics of participants.

Variables	Adherent	Non-Adherent	χ2	df	*p* Value *
	n (%)	n (%)			
Age (years)					
18–39	72 (61.0)	46 (39.0)	0.669	2	0.716
40–59	25 (54.3)	21 (45.7)			
≥60	13 (61.9)	8 (38.1)			
Gender					
Male	50 (66.7)	25 (33.3)	2.718	1	0.09
Female	60 (54.5)	50 (45.5)			
Race					
Malay	75 (56.0)	59 (44.0)	4.126	3	0.248
Chinese	15 (75.0)	5 (25.0)			
Indian	18 (62.1)	11 (37.9)			
Others	2 (100)	0 (0.0)			
Marital					
Married	69 (61.6)	43 (38.4)	4.637	2	0.098
Single	41 (58.6)	29 (41.4)			
Divorced	0 (0.0)	3 (100)			
No of children					
0	44 (57.1)	33 (42.9)	0.488	2	0.784
≤2	39 (62.9)	23 (37.1)			
>2	27 (58.7)	19 (41.3)			
Pets					
Absent	77 (60.2)	51 (39.8)	0.084	1	0.772
Present	33 (57.9)	24 (42.1)			
Duration of AR and use of INCS					
>5 years	64 (75.3)	21(24.7)			0.963
≤5 years	75(75.0)	25(25.0)	0.0021	1	

Notes: * Chi-squared test. Abbreviations: AR, allergic rhinitis; INCS, intranasal corticosteroids.

**Table 2:** The level of education, occupation and working hours.

Variables	Adherent	Non-Adherent	χ2	df	*p* Value *
	n (%)	n (%)			
Education level					
Primary	0 (0.0)	3 (100)	5.775	2	0.056
Secondary	44 (55.7)	35 (44.3)			
Tertiary	66 (64.1)	37 (35.9)			
Occupation					
Professional	47 (65.3)	25 (34.7)	1.655	1	0.198
Non-professional	63 (55.8)	50 (44.2)			
Working hours (per week)					
≤42 h	69 (60.0)	46 (40.0)	0.037	1	0.848
>42 h	41 (58.6)	29 (41.4)			

Notes: * Chi-Square test.

**Table 3:** Comorbid medical conditions and use of medicines.

Variables	Adherent	Non-Adherent	χ2	df	*p* Value *
	n (%)	n (%)			
BA					
Absent	86 (57.3)	64 (42.7)	1.487	1	0.223
Present	24 (68.6)	11 (31.4)			
Eczema					
Absent	101 (60.1)	67 (39.9)	0.330	1	0.566
Present	9 (52.9)	8 (47.1)			
BA and eczema					
Absent	82 (58.2)	59 (41.8)	0.418	1	0.518
Present	28 (63.6)	16 (36.4)			
DM					
Absent	103 (60.9)	66 (39.1)	1.793	1	0.181
Present	7 (43.8)	9 (56.3)			
HPT					
Absent	98 (60.1)	65 (39.9)	0.250	1	0.617
Present	12 (54.5)	10 (45.5)			
DM and HPT					
Absent	96 (60.8%)	62 (39.2%)	0.759	1	0.384
Present	14 (51.9%)	13 (48.1%)			
Use of medicines					
1–3	104 (60.5%)	68 (39.5%)			0.311
>3	6 (46.2%)	7 (53.8%)	1.027	1	

Notes: * Chi-squared test. Abbreviations: BA, bronchial asthma; DM, diabetes mellitus; HPT, hypertension.

**Table 4:** Post-hoc analysis of significant factors affecting medication adherence to intranasal corticosteroids.

Factors	B	S.E.	Wald	Crude OR	95% Confidence Interval		*p* Value *
					Lower	Upper	
House dust mite (Type DP)							
Present	0.755	0.336	5.052	2.13	1.101	4.11	0.001
Absent				1			
IgE level (UI/mL)							
IgE Elevated	0.991	0.349	8.08	2.69	1.36	5.34	0.004
IgE not Elevated				1			
Severity of TNSS at first visit							
Mild				1			0.001
Moderate	1.395	0.375	13.86	4.03	1.935	8.406	
Severe	2.716	0.499	19.65	15.13	5.689	40.215	
Severity of TNSS at second visit							
Mild				1			0.217
Moderate	0.887	0.718	1.525	2.427	0.594	9.922	
Severe	0.127	0.738	0.030	0.881	0.207	3.742	

Notes: * Simple logistic regression. Abbreviations: IgE, Immunoglobulin E; DP, *Dermatophagoides pteronyssinus*; TNSS, total nasal symptom score.

**Table 5:** The medication adherence to intranasal corticosteroids as evaluated by the Brief Medication Questionnaire.

	Adherent	Non-Adherent	χ2	df	*p* Value *
	n (%)	n (%)			
Regimen					
0	81 (100)	0 (0.0)	98.241	1	0.000
≥1	29 (27.9)	75 (72.1)			
Belief					
0	107 (60.8)	69 (39.2)			0.162
≥1	3 (33.3)	6 (66.7)	2.679	1	
Recall					
0	96 (60.8)	62 (39.2)			0.384
≥1	14 (51.9)	13 (48.1)	0.759	1	

Notes: * Chi-squared test.

**Table 6:** Barriers towards adherence to intranasal corticosteroids use.

**Regimen**	**Frequency (n)**
Symptoms not bothering	57
Run out of medication supply	5
Use when symptoms are bad	19
Missed doses due to fear of adverse effects	7
**Belief**	**Frequency (n)**
Experiencing bothersome effects	
-sneezing	1
-nasal obstruction	2
-dizziness	1
-after taste	2
Ineffective response	1
**Recall**	**Frequency (n)**
Taking too many medications	1
Busy schedule	17

## Data Availability

Data is available upon reasonable request from the author.

## Supplementary Materials

The following supporting information can be downloaded at: https://www.mdpi.com/article/10.3390/pharmaceutics14112459/s1, Figure S1: Patient’s diary. Table S1: Study proforma for data collection. Table S2: Atopic status, sensitization to allergens, serum IgE level and type of nasal spray. Table S3: Severity of total nasal symptoms score.
