# Supplementary material for: Medication Adherence to Intranasal Corticosteroids in Allergic Rhinitis Patients with Comorbid Medical Conditions
Source: Pharmaceutics. 2022 Nov 15;14(11):2459. doi: 10.3390/pharmaceutics14112459 (PMC9698755; doi:10.3390/pharmaceutics14112459)
Supplement: Supplementary file 1 [file pharmaceutics-14-02459-s001.zip › pharmaceutics-1958815-supplementary.pdf]

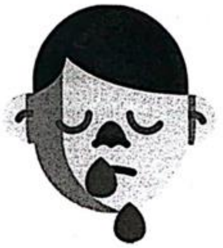

**The Study of Allergic Rhinitis patients adherence to intranasal corticosteroids**

**Name:** ____________________
**IC No:** ____________________
**Phone:** ____________________
**Email:** ____________________

## Contact Us

If you have any questions or doubts please do not hesitate to call us:

Phone: 012-3462945 / 03-26155555 ext: 1062
Email: prempreet_kaur84@yahoo.com

## DIARY

| DAY | WEEK 1 | WEEK 2 | WEEK 3 | WEEK 4 | Side effects |
|---|---|---|---|---|---|
| MON | | | | | |
| TUE | | | | | |
| WED | | | | | |
| THU | | | | | |
| FRI | | | | | |
| SAT | | | | | |
| SUN | | | | | |

| DAY | WEEK 5 | WEEK 6 | WEEK 7 | WEEK 8 | Side effects |
|---|---|---|---|---|---|
| MON | | | | | |
| TUE | | | | | |
| WED | | | | | |
| THU | | | | | |
| FRI | | | | | |
| SAT | | | | | |
| SUN | | | | | |

| DAY | WEEK 9 | WEEK 10 | WEEK 11 | WEEK 12 | Side effects |
|---|---|---|---|---|---|
| MON | | | | | |
| TUE | | | | | |
| WED | | | | | |
| THU | | | | | |
| FRI | | | | | |
| SAT | | | | | |
| SUN | | | | | |

COMMENTS:

____________________________________________
____________________________________________
____________________________________________
____________________________________________
____________________________________________
____________________________________________
____________________________________________
____________________________________________

Supplementary Figure S1. Patient’s diary.

Supplementary Table S1. Study proforma for data collection.

Please answer all the questions below. Fill in the space or tick (√) your answer in the box.

DATA COLLECTION SHEET

STUDY ID: _______________

AGE: ___________years old

GENDER: □ Male □ Female

RACE: □ Malay □ Chinese □ Indian □ Others: _______________

MARITAL: □ Single□ Married □ Divorced

DO YOU HAVE ANY CHILDREN: □ Yes, No of children: ____ □ No

EDUCATIONAL LEVEL: □ PMR □ SPM □ Certificate □ Diploma
□ Degree □ Master/PHD

OCCUPATION: _____________________

NO OF WORKING HOURS PER WEEK: □ <45hours □ >45hours

SMOKER: □ Yes □ No

DO YOU HAVE PETS AT HOME? □ Yes □ No

-what kind of pet? ____________

-number of pets? _____________

UNDERLYING MEDICAL ILLNESS: __________________

FOOD ALLERGY: □ Yes, what food: ________________ □ No

DRUG ALLERGY: □ Yes, what drug: ________________ □ No

HISTORY OF NASAL SURGERY: □ Yes, ____________ (Year) □ No

FAMILY HISTORY OF ALLERGY: □ Yes, to _____________ □ No

FAMILY HISTORY OF ASTHMA/RHINITIS/ECZEMA: □ Yes, ___________ □ No

USE OF MULTIPLE DRUGS, □ Yes, ___________□ No

Supplementary Table S2. Atopic status, sensitization to allergens, serum IgE level and type of nasal spray.

| **Variables** | **Adherent n (%)** | **Non- adherent n (%)** | **$X^2$** | **df** | ***P* value*** |
|---|---|---|---|---|---|
| Known allergy | | | | | 0.819 |
| Absent | 34 (60.7) | 22 (39.3) | 0.052 | 1 | |
| Present | 76 (58.9) | 53 (41.1) | | | |
| Family history | | | | | 0.260 |
| Absent | 79 (62.2) | 48 (37.8) | 1.267 | 1 | |
| Present | 31 (53.4) | 27 (46.6) | | | |
| Dust mites | | | | | 0.214 |
| Absent | 17 (50.0) | 17 (50.0) | 1.546 | 1 | |
| Present | 93 (61.6) | 58 (38.4) | | | |
| Type of dust mites | | | | | |
| DP | | | 5.149 | 1 | 0.023 |
| Absent | 23 (46.0) | 27 (54.0) | | | |
| Present | 87 (64.4) | 48 (35.6) | | | |
| DF | | | 0.867 | 1 | 0.352 |
| Absent | 51 (56.0) | 40 (44.0) | | | |
| Present | 59 (62.8) | 35 (37.2) | | | |
| Seafood | | | | | 0.272 |
| Absent | 57 (55.9) | 45 (44.1) | 1.207 | 1 | |
| Present | 53 (63.9) | 30 (36.1) | | | |
| Cat dander | | | | | 0.242 |
| Absent | 83 (57.2) | 62 (42.8) | 1.369 | 1 | |
| Present | 27 (67.5) | 13 (32.5) | | | |
| Cockroach | | | | | 0.348 |
| Absent | 72 (57.1) | 54 (42.9) | 0.880 | 1 | |
| Present | 38 (64.4) | 21 (35.6) | | | |
| Type of nasal spray | | | | | 0.127 |
| Budesonide | 54 (53.5) | 47 (46.5) | 4.122 | 2 | |
| Mometasone | 30 (62.5) | 18 (37.5) | | | |
| Fluticasone | 26 (72.2) | 10 (27.8) | | | |
| IgE level (UI/ml) | | | | | 0.004 |
| Normal | 19 (41.3) | 27 (58.7) | 8.371 | 1 | |
| Elevated | 91 (65.5) | 48 (34.5) | | | |

**Notes:** *Chi-Square test.

**Abbreviations:** IgE, Immunoglobulin E; DP, dermatophagoides pteronyssinus; DF, dermatophagoides farinae

Supplementary Table S3. Severity of total nasal symptoms score.

| Severity of TNSS | Adherent n (%) | Non- adherent n (%) | $X^2$ | df | *P* value* |
|---|---|---|---|---|---|
| 1st visit | | | | | 0.000 |
| Mild | 17 (29.8) | 40 (70.2) | 37.016 | 2 | |
| Moderate | 48 (63.2) | 28 (36.8) | | | |
| Severe | 45 (86.5) | 7 (13.5) | | | |
| 2nd visit | | | | | 0.006 |
| Mild | 50 (49.0) | 52 (51.0) | 10.304 | 2 | |
| Moderate | 53 (72.6) | 20 (27.4) | | | |
| Severe | 7 (70.0) | 3 (30.0) | | | |
| 3rd visit | | | | | 0.085 |
| Mild | 75 (54.7) | 62 (45.3) | 4.921 | 2 | |
| Moderate | 33 (73.3) | 12 (26.7) | | | |
| Severe | 2 (66.7) | 1 (33.3) | | | |

**Notes:** *Chi-Square test.
**Abbreviations:** TNSS, total nasal symptom score
